# Bio-cleaning of nitrate salt efflorescence on stone samples using extremophilic bacteria

**DOI:** 10.1038/s41598-018-38187-x

**Published:** 2019-02-07

**Authors:** Ida Romano, Mario Abbate, Annarita Poli, Loredana D’Orazio

**Affiliations:** 1 Institute of Biomolecular Chemistry of Consiglio Nazionale delle Ricerche, 80078 Pozzuoli, Naples Italy; 2 Institute for Polymers, Composites and Biomaterials of Consiglio Nazionale delle Ricerche, 80078 Pozzuoli, Naples Italy

## Abstract

For the first time, we propose the use of an extremophilic bacterium to remove nitrate salt efflorescence from the surfaces of stone samples. A haloalkaliphilic bacterium was selected “ad hoc” for its ability to reduce nitrates; i.e. *Halomonas campaniensis* sp. nov., strain 5AG^T^ (DSM 15293^T^, ATCC BAA-966^T^). Quantitative monitoring of nitrate content, on untreated and treated surfaces of stone samples artificially enriched with nitrate, as a function of incubation/treatment time, was carried out by molecular spectroscopy. The results obtained reveal the good performance of *Halomonas campaniensis* bacterium in decreasing nitrate concentration on stone surfaces both in a controlled laboratory environment for temperature and relative humidity and in a real outdoor environmental conditions.

## Introduction

It is well known that degradation phenomena plaguing cultural heritage, such as deposition of organic substances, black crust formation and mineral salt crystallization, can be tackled by means of biotechnologies based on different cultures of viable bacteria^1–8^. A common thread among all the microorganisms used is that they live under conventional conditions referred as “normal” or “physiological”, because such microorganisms grow and thrive under conditions which are comfortable also for human beings: i.e. moderate temperature close to 37 °C, pH near neutral value, salinity ranging from 0.9% to 3.0%, water availability, oxygen presence, atmospheric pressure, with carbon and energy availability.

To remove, in particular, nitrate crusts from stony materials several sets of nitrate-reducing aerobic and facultative anaerobic bacteria, such as *Pseudomonas denitrificans, Pseudomonas stutzeri*, *Pseudomonas aeruginosa, Pseudomonas pseudoalcaligenes* and *Paracoccus denitrificans*, were used^9,10^. More or less satisfactory results were achieved depending upon a combination of interrelated factors; bacterial cell strains utilized, adopted protocol, type of carrier used, treatment duration, environmental conditions, as well as, own peculiar characteristics of the salt crusts. Bio-cleaning interventions aimed at the removal of nitrate layers has been carried out on the external walls of the cathedral of Matera, Italy and the wall paintings placed in the lunettes of the central vault of the Santo Juanes church in Valentia, Spain^11,12^. The external wall of the cathedral of Matera was treated with *Pseudomonas pseudoalcaligenes* cells entrapped in a Carbogel carrier and then covered with a PET film. *Pseudomonas stutzeri* DSMZ 5190 was applied on wall painting surfaces of the Santo Juanes church in Valentia by means of Japanese paper and agar carrier layers. Infrared heat lamps were used during the treatment to ensure the correct metabolic activity of the bacteria. Nevertheless, traditional cleaning methods of cultural heritage, based on chemical and mechanical treatments, are still preferred, microorganisms being generally regarded as dangerous agents. Hence, it follows that demonstrating the safety of bio-cleaning treatments is an essential step required for the adoption of bio-restoration methods for conservation of items of historic and cultural importance.

The present contribution therefore suggests to employ extremophilic microorganisms to perform bio-cleaning treatments on stone substrata as an alternative. Extremophiles are microorganisms that populate peculiar ecological niches characterized by very low or high temperatures, extreme values of pH, high salt concentration, high pressure or the presence of heavy metals or radiation. They belong to *Archaea, Bacteria* and *Eukarya* domains^13^. Deep sea hydrothermal vents and springs, polar regions, saltworks, nuclear reactors, crude oil and heavy metal polluted soils are some examples of habitats inhabit by extremophiles^14,15^. Since extremophiles thrive in extreme conditions, their molecules are often more stable than the mesophilic counterparts, providing industrially important sources of enzymes and biopolymers. Biotechnological applications of extremophiles have been, so far, outlined in fields such as pharmaceutical, medical, industrial, ecological and food^16–18^. With the aim of widening the applications fields of extremophilic microorganisms, and considering that there is a need to demonstrate clearly the complete safety of bio-cleaning treatments on cultural heritage materials, our investigation started from the following idea. Applications under normal conditions (without nutrients supporting additional growth) of microorganisms, which can only live in particular ecological niches, should at least limit possible interactions and effects on the environment, cultural heritage and restorers. In the case of extremophiles that do not survive or grow under “normal” conditions, it follows that no undesired metabolic processes are to be expected to occur on substrata during and after treatments. If so, environmental sustainability along with safe application and removal processes for both restorers and cultural heritage artifacts might be ensured. Hence, a screening of extremophiles with different phenotypes (thermophiles, psychrophiles, halophiles, haloalkaliphiles), belonging to the cell bank of the Institute of Biomolecular Chemistry (ICB) of Consiglio Nazionale delle Ricerche (C.N.R.), Pozzuoli, Italy was carried out to identify the most effective in carrying out the denitrification process. A haloalkaliphilic aerobic, non-pathogenic and non-sporulating bacterium *Halomonas campaniensis* strain 5AG^T^ was so selected for its comparatively higher ability to reduce nitrate^19^. The identification of this strain within our screen is consistent with literature, reporting that species of the genus *Halomonas* possess denitrification abilities such that it is to be considered an phylogenetic marker^20^.

*H. campaniensis* bacterium was first applied to stone samples consisting of tuff, specifically Neapolitan Yellow Tuff (NYT), artificially enriched with nitrate first in the lab under controlled conditions, and then under real environmental conditions. NYT is a very complex and heterogeneous volcanic rock, spread over a wide zone of the Campania Region (Southern Italy), named the Phlegraean Fields (from Greek “burned”), situated to the West of Naples including the Pozzuoli area. NYT was used as a building material since ancient times owing to its peculiar color, light weight, easy workability and good insulating properties. The basic agent of the weathering phenomena undergone by stone-built structures is mainly represented by water. Water action is manifested both directly through hydrolysis processes in silicates and dissolution in carbonates and indirectly through processes of hydration-dehydration, freeze-thaw cycles and crystallization of salts. The appearance of efflorescence caused by the evaporation of ascending waters that deposit soluble salts on the stone surface is one of the most frequent types of weathering in NYT. The presence of nitrates in ground water has its origin in the natural decomposition of organic nitrogenous materials (like proteins from plants, animals and so on) by microorganisms. The ammonium ion formed during that breakdown is oxidized to nitrite and nitrate following the biological oxidation process of nitrification. Nitratation decay of exposed stonework in polluted urban areas is also caused by atmospheric pollution^21^. NYT substrata have been previously used in works to carry out the first synthesis of poly(urethane urea) by *in situ* polymerization inside stone^22^ and to perform eco-sustainable protective treatments based on a water-dispersed titanium dioxide/poly(carbonate urethane) nanocomposite^23–25^. As for the previous work, NYT samples were used as substrata representative of stony materials that are complicated to protect. Determination of nitrate content on the surface of NYT samples artificially enriched with nitrate, before and after bio-cleaning treatments, was preliminary performed by means of the Aquamerck nitrate test. In order to achieve a more accurate nitrate determination Fourier Transform Infrared (FTIR) spectroscopy analysis was carried out. The main goal was to assess if the selected bacterium maintains its nitrate reducing ability when it is applied on stone substrata out of its ecological niche along with the safety and eco-sustainability with regard to its potential application as a bio-cleaning agent. In spite of the fact that the use of microorganisms is frequently connected with detrimental effects on the durability of cultural heritage materials^26–28^, the use of extremophilic bacteria to clean surfaces could establish the basis of a new branch of biotechnology applied to the conservation and restoration of cultural heritage.

## Results and Discussion

### Nitrate removal efficiency of extremophilic *Halomonas campaniensis* bacterium

Screening of bacterial nitrate reduction carried out by using Aquamerck nitrate tests showed that *H. campaniensis* exhibited comparatively higher ability to reduce nitrate, i.e. 1 g/l of KNO_3_ after 24 h (see Table 1). Results achieved by Gonzáles-Domenech^20^ proved that *H. campaniensis* reduces nitrate and nitrite aerobically and anaerobically. Its ability is due to the presence of nitrate and nitrite reductase enzymes responsible for the denitrification process, where nitrate and nitrite and gaseous NO and N_2_O act as terminal acceptors for electron transport in place of oxygen (O_2_).

**Table 1:** Selected microorganisms used to assess nitrate reduction after 24 h of growth along with related isolation place, growth media, optimal pH and temperature values.

Microorganisms	Isolation place	Growth media	Optimal pH values	Optimal temperature (°C)	Nitrate reduction after 24 h (g/l)	References
*Halomonas campaniensis*	Mineral pool in Malvizza, Avellino, Campania region, Italy	Medium A	9.0	35	1	^19^
*Bacillus saliphilus*	Mineral pool in Malvizza, Avellino, Campania region, Italy	Medium A	9.0	37	0.8	^43^
*Haloterrigena hispanica*	Salt lake in Fuente de Piedra, Spain	DSM 372	7.0	50	0.5	^41^
*Geobacillus toebii* subspecies *decanicus*	Hot compost, Pomigliano, Naples, Italy	TH medium	7.0	65	0.7	^42^
*Parageobacillus thermantarcticus*	Geothermal soil of Mt. Melbourne, Antarctica	YN medium	6.0	60	0.6	^39, 40^
*Nesterenkonia aurantiaca*	Cape King, Antarctica	Medium B	9.0	25	0.6	^44^

Aquamerck nitrate tests carried out on powder samples of NYT stone artificially enriched with nitrate, before and after treatment with *H. campaniensis*, showed that this microorganism retained encouraging potential for nitrate reduction when applied on stone substrata in normal conditions. An average salt concentration of 1.25 g/l was initially found on stone surfaces, whereas after a 3 h treatment at 35 °C and 93% humidity concentrations decreased to 0.50 g/l. Hence, a reduction of around 60% of starting nitrate concentration occurred.

The nitrate removal ability shown by *H. campaniensis* on stone was also determined through nitrite determination on NYT powder samples enriched with nitrate. Before bacteria application, the nitrite test carried out found a complete absence of nitrite, whereas after 3 hours of incubation/treatment an average nitrite concentration considerably lower (0.23 g/l) than that of the reduced nitrate was found, suggesting that the nitrite formed through *H. campaniensis* denitrification undergoes further reduction to volatiles.

**Figure 1:**
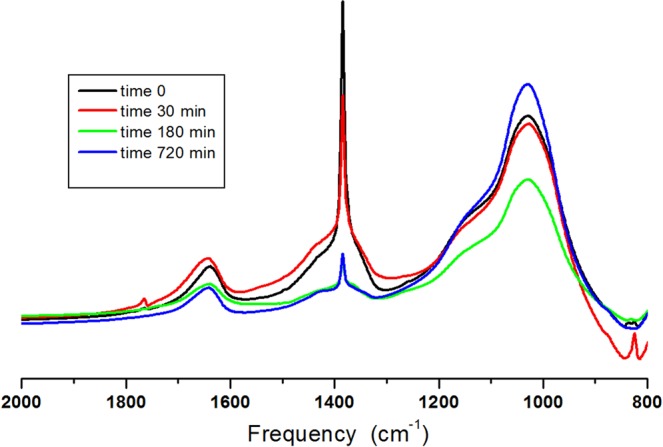
Typical FTIR spectra of NYT samples before and after bio-cleaning treatments.

The qualitative Aquamerck nitrate determination on stone was then refined by means of FTIR spectroscopy analysis. Typical FTIR spectra recorded, before and after bio-cleaning performed on lab scale, are shown in Fig. 1. FTIR spectra exhibited by plain NYT (Supplementary Fig. S1) and FTIR spectra exhibited by plain Potassium nitrate (Supplementary Fig. S2) were taken as a references for spectral analysis. From the literature^29,30^, NYT contains several zeolitic phases, in particular chabasite and phillipsite, consisting of hydrated Al-silicates. Hence, NYT typical FTIR spectra show the most intense vibration of Al-silicates at 1635 cm^−1^ and 992 cm^−1^ together with hydrogen bonding of the OH- at 3300 cm^−1^ and water vibration at 3600 cm^−1^ ^31^. Potassium nitrate is an ionic salt consisting of Potassium cations and Nitrate anions can therefore be identified through characteristic peaks of planar ions having trigonal symmetry active in the infrared. There are four fundamental modes of molecular vibration: symmetric stretching (ν_1_) IR inactive_,_ out of plane bending (ν_2_)_,_ doubly degenerate anti-symmetric stretching (ν_3_) and doubly degenerate planar bending (ν_4_)^32^. Frequencies and assignments of the main absorption bands of Potassium nitrate used to enrich NYT samples can be found in Supplementary Table S1. A comparison of spectra between Potassium nitrate and NYT reveals that nitrate molecular vibration caused by anti-symmetric stretching (ν_3_) falls on a spectral range where no absorption ascribed to NYT is observed; the corresponding peak is well resolved and centred at 1385 cm^−1^. As expected, typical FTIR spectra shown by nitrate enriched NYT samples exhibit also a nitrate absorption band centred at 1385 cm^−1^ (Supplementary Fig. S3). As shown in Fig. 1, typical FTIR spectra recorded, before and after bio-cleaning treatments, clearly reveal that the height of the nitrate peak centred at 1385 cm^−1^ decreases with increasing the incubation/treatment time; the registered corresponding absorbance values reducing with increasing incubation/treatment time. This finding indicates that stone nitrate efflorescence may be affected by *H. campaniensis*. It should be pointed out that such a peak, in addition to being free of interference by the stony phase, shows a strong enough change in intensity as a function of nitrate amount that it was found to be appropriate for carrying out nitrate quantitative analysis by applying the Lambert-Beer law^33,34^. Potassium nitrate calibration spectra were so accomplished analysing salt samples weighted by means of a Gibertini analytical balance. The absorbance values recorded at 1385 cm^−1^ normalized for sample thickness (reduced absorbance) against nitrate amount in milligrams were plotted to construct a calibration plot (Supplementary Fig. S4). The straight line achieved shows a value for the coefficient of determination (R^2^) as high as 0.99 and a standard error as low as 0.05 representing the validity of the determination method. Nitrate amounts on stone surfaces were therefore evaluated by measuring the absorbance value at 1385 cm^−1^, calculating the reduced absorbance value and identifying the corresponding nitrate amount through the equation of the straight calibration line.

To exemplify the nitrate determination method used, Table 2 reports for plain NYT samples and NYT samples treated with *H. campaniensis* for 3 h the following values: KBr pellet thickness (mm), peak height at 1385 cm-1 (a.u.), peak height at 1385 cm-1 normalized by pellet thickness (a.u./mm), nitrate amount determined through the calibration plot (mg) and nitrate amount (mg) contained in 1 g of stone powder.

**Table 2:** Values of pellet thickness (mm), peak height at 1385 cm^−1^(a.u.), peak height at 1385 cm^−1^ normalized by pellet thickness (a.u./mm), nitrate amount determined through a calibration plot (mg) and nitrate amount (mg) contained in 1 g of NYT powder obtained from plain NYT samples (a); 1 g of NYT powder obtained from NYT samples treated under controlled conditions with *Halomonas campaniensis* for 3 h (b); 1 g of NYT powder obtained from NYT samples treated with *Halomonas campaniensis* outdoors for 3 h (c).

Pellet thickness (mm)	Peak height at 1385 cm^−1^(a.u.)	Peak height at 1385 cm^−1^normalized by pellet thickness (a.u./mm)	Nitrate amount determined through calibration plot (mg)	Nitrate amount (mg) contained in 1 g of NYT powder
**(a) Values referred to 1 g of NYT powder achieved for plain NYT samples**				
1.140	1.155	1.012	1.198	799
1.140	1.150	1.009	1.193	795
1.170	1.306	1.116	1.319	880
1.140	1.137	0.997	1.179	786
1.440	1.262	0.874	1.033	861
1.140	1.153	1.011	1.195	797
**(b) Values referred to 1 g of NYT powder achieved for NYT samples treated under controlled conditions with** ***Halomonas campaniensis*** **for 3 h**				
1.100	0.167	0.152	0.179	179
1.141	0.261	0.229	0.271	180
1.160	0.348	0.300	0.355	237
1.130	0.189	0.163	0.193	193
1.141	0.261	0.229	0.271	180
1.160	0.348	0.300	0.355	179
**(c) Values referred to 1 g of NYT powder achieved for NYT samples treated with** ***Halomonas campaniensis*** **outdoor for 3 h**				
1.184	0.985	0.832	0.980	490
1.240	0.473	0.301	0.356	223
1.153	0.680	0.589	0.697	410
1.229	0.980	0.798	0.943	428
1.227	0.285	0.232	0.274	196
1.150	0.673	0.585	0.692	461

**Figure 2:**
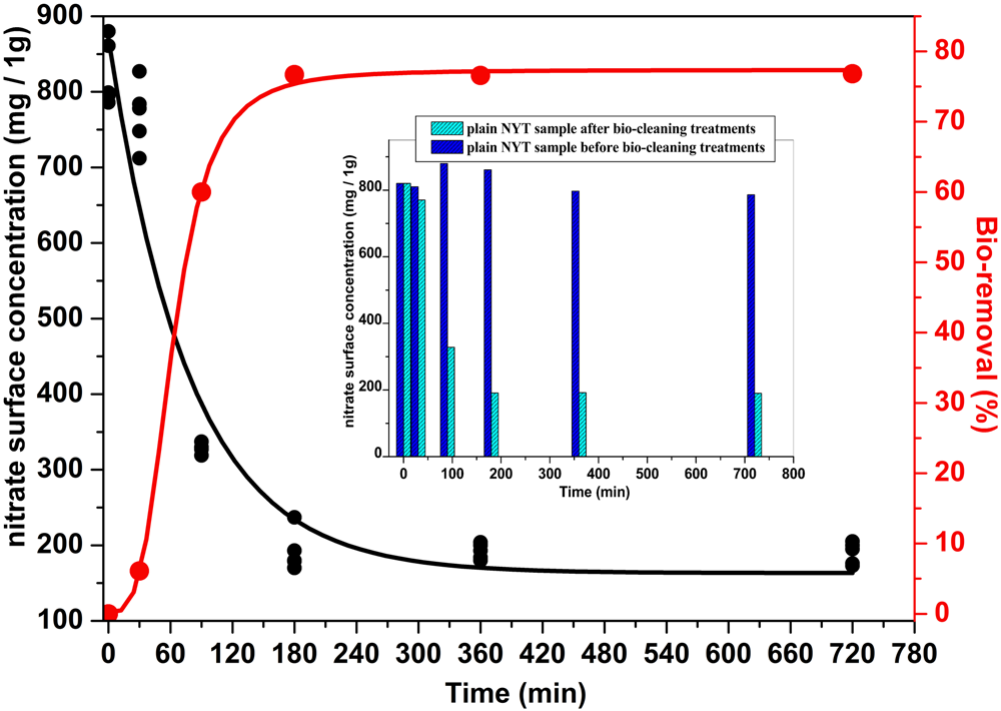
Nitrate surface concentration (mg/g) as a function of incubation/treatment time; Efficiency of extremophilic *Halomonas campaniensis* (%) as a function of time of treatment.

By plotting the values of reduced absorbance as a function of time it can be observed that absorbance values decrease with increasing time approaching a plateau. The efficiency of the microorganism, expressed both as a bio-removal percentage and nitrate surface concentration (mg/g), is shown in Fig. 2 as a function of time of treatment. Nitrate surface concentration (mg/g) exhibited by control samples was also compared with that achieved after bio-cleaning (Fig. 2). The bio-removal data were fitted by the Hill sigmoid equation with a R^2^ value equal to 0.9993. The nitrate surface concentrations were fitted by an exponential equation with an R^2^ value equal to 0.9403 and non-linear regression analysis showed that the results achieved were statistically significant (P < 0.0001). As shown, for the experimental conditions set up at least, optimal time of treatment is 3 hours with a nitrate bio-removal equal to 77 ± 3 (%) of nitrate amount deposited on the surface of the NYT samples; such a percentage is comparable to that reported in the literature^11,12^. No further efficiency increase was observed with increasing time of treatment. Before treatment, the nitrate concentration on stone surfaces was equal to 820 ± 40 mg/g, whereas after the concentration was lowered to 191 ± 26 mg/g by *H. campaniensis* (see Fig. 2) thus revealing its significant ability to convert nitrate also on stony substrata.

It is interesting to note the advantages of using *H. campaniensis* for nitrate removal rather than the microorganisms reported in the literature, such as *Pseudomonas* strains, etc… and*/*or non-biological methods. Since the introduction of the biological approach to the protection of cultural heritage, private and public sector restoration committees asked if such an approach is potentially hazardous and still demand to the scientific community a clear information about the safety of this biotechnologies. The absence of risk is therefore the key factor for spreading out bio-treatments on cultural heritage^35^. For nitrate bio-removal, non-pathogenic *Pseudomonas stutzeri* strains have been mainly used, even though these species are known to be opportunistic, naturally transformable and in some cases pathogenic^36^. The use of extremophilic microorganisms can conversely ensures the safety of interventions in the absence of risk, because they need growth conditions to proliferate that do not correspond to those of most living beings. It should also be marked the following. After treatment, no *H. campaniensis* viable cells are able to survive as proved by the results below described. Hence, no negative effects due to metabolism of viable cells potentially remained on the treated items are to be expected, indicating that *H. campaniensis* is comparatively safer also for cultural heritage. As a result, the current need for short, medium and long-term monitoring after treatment, which is essential to determine the absence of viable cells, could be eliminated with significant reduction of both treatments duration and costs. Furthermore the nitrate removal percentage by *H. campaniensis* was achieved without setting up of multilayer bio-systems and/or devices application and/or water based methods as reported elsewhere^11,12^. Nitrate cleanings by the extremophilic microorganism tested can be considered environmentally friendly and therefore advantageous also in comparison with physico-chemical methods, that generally require the use of solvents, excessive removal of the original material, etc… In particular water based methods to remove nitrate can damage the stone because nitrate can be transferred in the deep by water.

**Figure 3:**
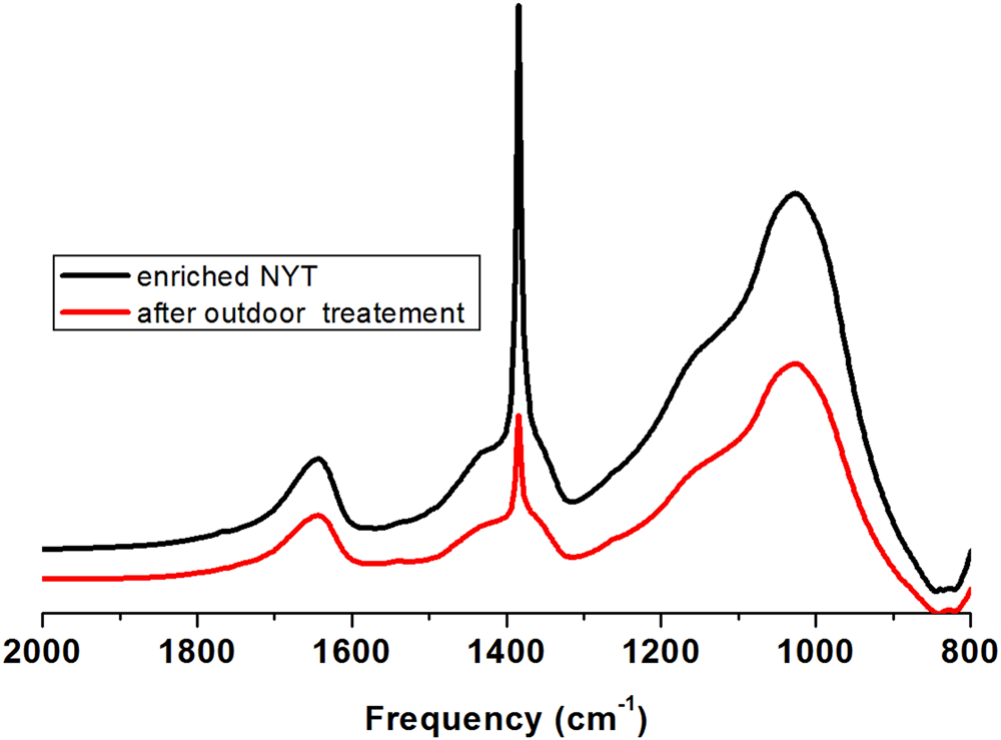
Typical FTIR spectra of NYT samples outdoor exposed before and after bio-cleaning treatments.

Figure 3 shows typical FTIR spectra recorded on enriched NYT samples before and after bio-cleaning treatments performed outdoors in Pozzuoli. As shown, notwithstanding that the parameters required for *H. campaniensis* thriving, such as salinity, temperature and relative humidity, were not optimal, the height of the nitrate peak centred at 1385 cm^−1^ was found reduced after a treatment time of 3 h. This suggests that *H. campaniensis* under “normal” conditions maintains the ability to convert nitrate for 3 h at least. From data reported in Table 2 an average bio-removal percentage equal to 55 ± 15 (%) can be calculated to be reasonably related to the exposure temperature, humidity and other environmental outdoor conditions such as oxygen level, radiation, etc… affecting the bacterial activity. It cannot be excluded that this value, lower than that achieved with the lab, could be increased by selecting more appropriate outdoor exposure conditions and/or protracting treatment time. It should also be considered that the physical properties of the efflorescence used in these tests may be different from those of the historical monuments to be restored.

### Interaction between *Halomonas campaniensis* and real environments

NYT stone samples treated with and without *H. campaniensis* were checked at the established times after exposure to the outdoor Pozzuoli environment. All the washings carried out as indicated in Materials and Methods section showed microorganism growth in sterile medium A after an incubation of 48 h in optimal conditions of *H. campaniensis* growth. It is to be noted that samples tested for the presence of *H. campaniensis* after 1, 3 and 7 exposure days exhibited variable amount of both rod-shaped and cocci-shaped bacteria; cocci-shaped bacteria occurring singly, in pairs or in irregular clusters. The morphology and length (about 2–3 μm) of the rod-shaped bacteria corresponded approximately to that shown by *H. campaniensis* after 24 h of incubation in optimal growth conditions^19^. As far as cocci-shaped bacteria, from Bergey’s Manual of Systematic Bacteriology^37^ these microorganisms can belong to several different genera. Considering that the outdoor exposure was carried out in a marina, and that the medium A, used for the washings contained 10% of NaCl, it could be hypothesized that the cocci-shaped bacteria observed belong to Gram positive genera such as *Marinococcus, Planococcus, Salinicoccus*, etc. The species of such genera are, in fact, widely distributed in marina habitats and saline soil and grow at the optimum temperature range of 25–37 °C requiring 0–20% of NaCl (*Marinococcus*), 1–15% (*Planococcus*) and 1–25% (*Salinicoccus*). In particular, after 1 day of exposure, the optical field observed through phase-contrast microscopy consisted overall of rod-shaped bacteria and some cocci-shaped bacteria with an average size ranging between 0.5–2 μm. All of the rod-shaped bacteria were Gram negative (as expected by *H. campaniensis*), while the cocci-shaped bacteria were Gram positive. These differences in the Gram staining for rod and cocci-shaped microorganisms were observed at all times tested. After 3 days of exposure, the amount of rod-shaped bacteria observed was about 80% and cocci-shaped bacteria about 20%. After 7 days of exposure, the optical field consisted of about 30% rod-shaped and 70% cocci-shaped bacteria. At 14, 21 and 30 days only cocci-shaped bacteria were present in the growth, i.e. the rod-shaped microorganisms, attributable to *H. campaniensis*, were no longer present. These findings indicated that with increasing exposure time proliferation of rod-shaped bacteria decreases and they subsequently disappear while cocci-shaped bacteria prevail.

It is worthnoting that the washings recovered from stone samples exposed without *H. campaniensis* showed that after 72 h of incubation, for all the investigated exposure times, only cocci-shaped bacteria were present, suggesting that such bacteria are predominant in the outdoor exposure environment. Hence, for the outdoor environmental conditions tested at least, *H. campaniensis* is unable to grow likely due to out-competition by microorganism species present in the exposure environment.

Washing tests conducted on treated and untreated stone samples indoor exposed within the ICB microbiology lab in Pozzuoli, respectively showed, after 48–72 h of incubation, growth of rod-shaped bacteria with morphological features close to that shown by *H. campaniensis*, but comparatively longer and no growth.

### Removal efficiency of *Halomonas campaniensis* after washing

**Figure 4:**
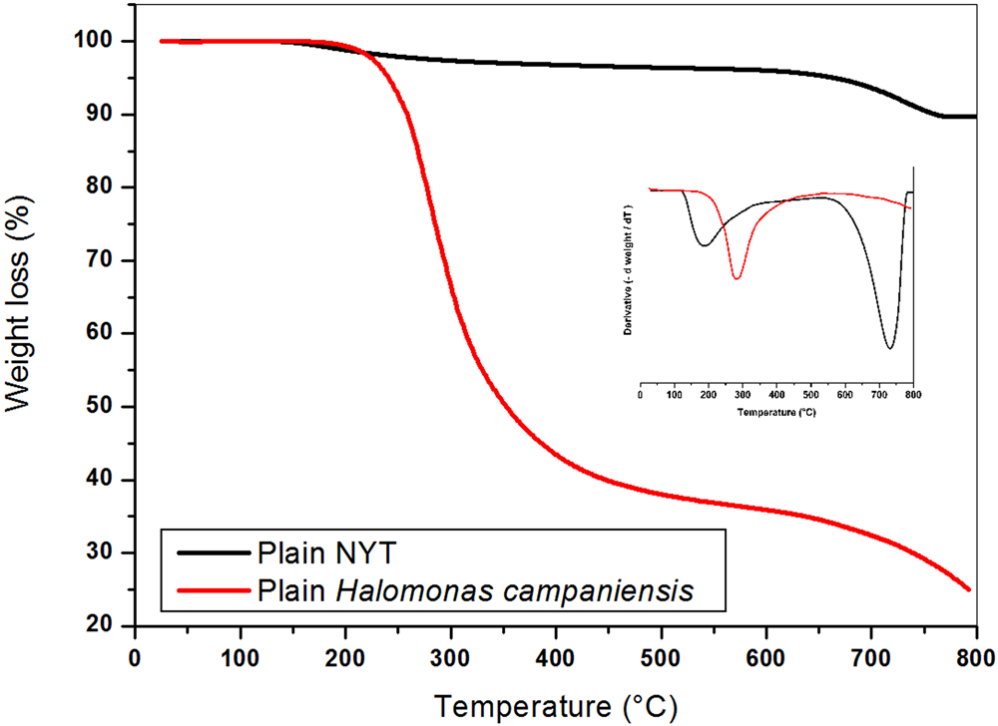
Typical TGA curves shown by: plain NYT (**a**) and plain *Halomonas campaniensis* specimen (**b**).

The structure and mineralogical composition of NYT stone affect free water evaporation and dehydration processes undergone with increasing temperature as revealed by TGA analysis. As shown in Fig. 4 weight loss corresponding to water loss occurs up to 400 °C; this feature is due to the presence of zeolites that are able to strongly retain water^30^. It was calculated that a 3.2% weightless of stone initial weight is exhibited for temperatures ranging between 100 °C and 400 °C (T_d_ max 188 °C). A comparatively higher weight loss equal to 6.3% of initial weight starts at 600 °C, ascribed to deidroxylation reactions undergone by smectite (Td max 734 °C); i.e to loss of hydroxyl water. The occurrence of such reactions was confirmed by FTIR spectra of NYT samples after heating up to 900 °C (Supplementary Fig. S5). As shown by Fig. S5, after heating, the OH stretching peak ranging between 3300 cm^−1^ and 3600 cm^−1^ disappears and the height of the Al-silicates peaks at 1635 cm^−1^ and 992 cm^−1^ are strongly reduced.

KNO_3_ salt decomposition starts at temperatures higher than its boiling point, which is 400 °C^38^. The thermal decomposition of alkali metal nitrate salts exposed to high temperature occurs on the basis of three different mechanisms: nitrite formation in the melt and oxygen release, alkali metal oxide formation in the melt and nitrogen or nitrogen oxide release, and vaporization of the nitrate salts. The first type of reaction releases gaseous oxygen as a decomposition product. The secondary decomposition reaction is rather complicated by an overlapping of different effects such as the decomposition of the intermediate nitrite formed, caused by its lower decomposition temperature, and the formation of oxides in the melt^38^. Nitrate salt used in the present work was found durable up to a minimum of 500 °C.

The TGA curve achieved for *H. campaniensis* cells shows two degradation steps (see Fig. 4). The first one occurs between approximately 200 °C and 600 °C (T_d_ max 280 °C) releasing 64% of volatiles; the second in the temperature range from around 600 °C to 800 °C releasing 10% of volatiles. Both weight losses are related to decomposition of complex patterns of products formed under conditions of intermediate pyrolysis, such as hydrocarbon chains of fatty acids and polysaccharides components of the major bacterial constituents, proteins and nucleic acids. It is to be noted that the highest weightless (56%) occurs between 200 °C and 400 °C.

**Figure 5:**
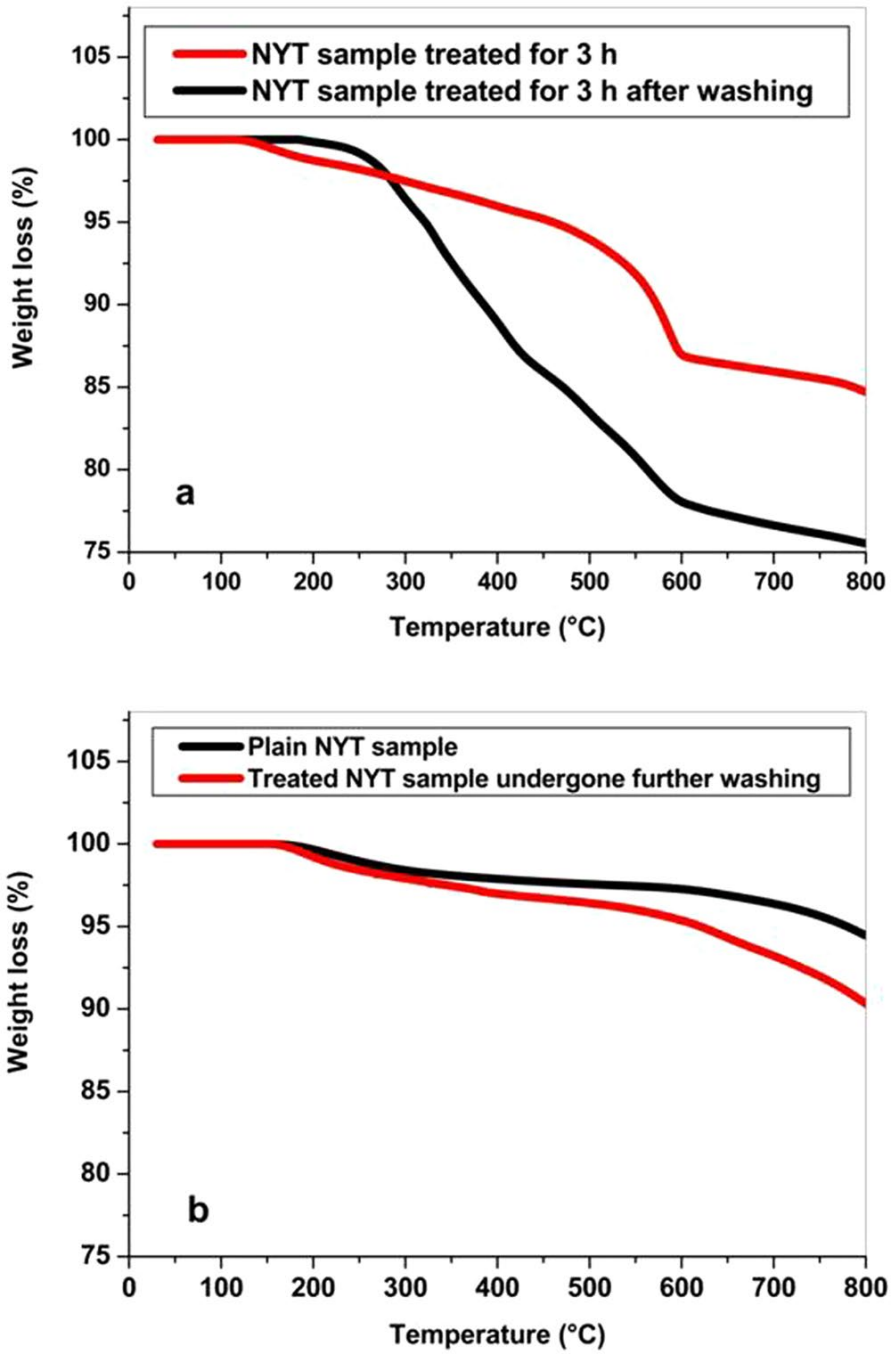
Typical TGA curves shown by NYT samples: (**a**) treated for 3 h before and after washing; (**b**) plain and treated undergone further washing cycle.

Figure 5a shows a typical degradation curve (% residual weight against temperature) exhibited by nitrate enriched NYT samples treated with *H. campaniensis* for an incubation/treatment time of 3 hours. As shown, two degradation steps can be clearly detected as a function of temperature. An approximately 22% weightless is found for temperatures ranging between 200 °C and 600 °C; whereas 2.5% weightless occurs between 600 °C and 800 °C. Taking into account that nitrate decomposition starts at temperatures higher than 500 °C, the weight loss observed for temperatures between 200 °C and 400 °C is to be ascribed to loss of water retained by tuff zeolites (2% TGA evaluated) and volatiles loss due to bacterial degradation. Figure 5a also shows a typical degradation curve obtained analysing NYT samples treated with *H. campaniensis* for 3 h and subsequently undergoing washing by water. It is interesting to note that, for temperatures between 200 °C and 600 °C, the TGA curve for NYT samples washed with water after *H. campaniensis* bio-cleaning for 3 hours shows a quite different profile from that shown by the same NYT samples unwashed. A comparatively lower weight loss than that found for unwashed stone samples is in fact observed. In particular, between 200 °C and 400 °C the unwashed and washed samples lose 10.8% and 2.8% of their initial weight respectively. Considering a 2% weight loss undergone by NYT in such a temperature range, it could be inferred that for the washed samples only 0.8% weight loss could be ascribed to bacterial presence. Typical TGA profiles exhibited by samples undergoing further washing cycles with water can be superimposed to that shown by plain tuff (see Fig. 5b), thus suggesting an absence of organic material.

By standard plate count methods, ten washings of NYT treated surfaces with a sterile sponge impregnated with 10 ml of sterile distilled water ensured 99.99% of bacterial removal. The monitoring of *H. campaniensis* viability, carried out after three months, showed complete absence of bacterial cells.

**Figure 6:**
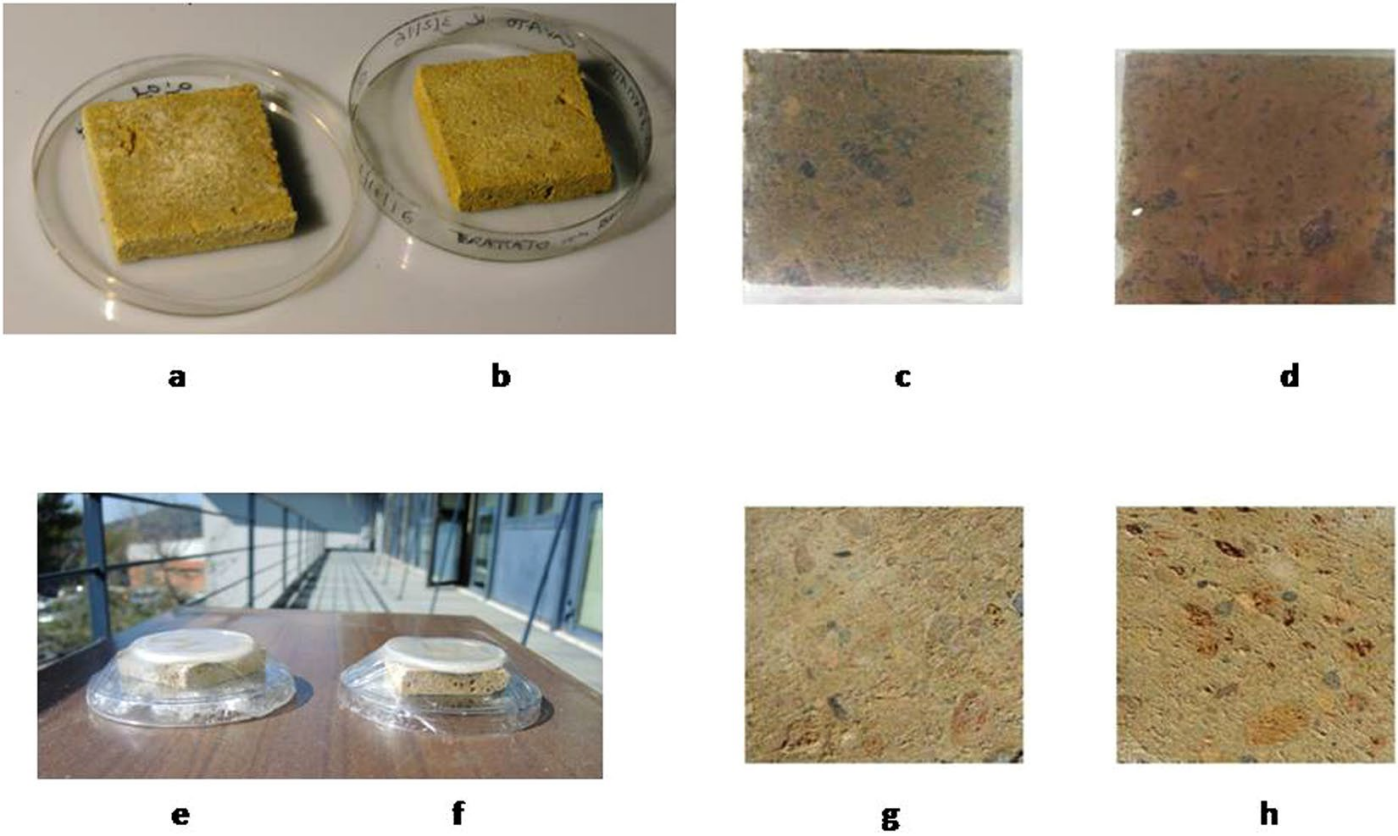
Typical photomicrographs of: (**a**) NYT samples after nitrate enrichment; (**b**) NYT samples before nitrate enrichment; (**c**) NYT samples surface supplemented with agar disc untreated; (**d**) NYT samples surface supplemented with agar disc treated with *Halomonas campaniensis*; (**e**) NYT sample outdoor exposed supplemented with agar disc untreated; (**f**) NYT sample outdoor exposed supplemented with agar disc treated with *Halomonas campaniensis*; (**f**) NYT sample treated surface after removal of *Halomonas campaniensis* specimen by spatula; (**g**) NYT sample treated surface after removal of *Halomonas campaniensis* specimen by spatula and subsequent water washing by sponge.

Also of note is that no chromatic alterations were observed by visual inspection on the surface of NYT samples treated with the microorganism (see Fig. 6g,h).

## Materials and Methods

### Screening of extremophilic bacteria with nitrate reductase enzyme

The Institute of Biomolecular Chemistry (ICB) of Consiglio Nazionale delle Ricerche (C.N.R.) in Pozzuoli (Naples), Italy possesses a large cell bank that contains interesting extremophilic microorganisms isolated in different ecological niches present in the world. The first step was to test the capability of extremophiles present in the ICB-cell bank to reduce nitrate. The following species were checked:

*Halomonas campaniensis*^19^ an aerobic, motile rod-shaped Gram negative staining bacterium. It is a haloalkaliphilic species isolated from samples of water with algal mat collected in a mineral pool in Malvizza (Campania–Italy), was grown in the following medium A (pH 9) containing (per litre): 10 g yeast extract, 3 g trisodium citrate, 2 g KCl, 1 g MgSO_4_•7H_2_O, 100 g NaCl, 0.36 g MnCl_2_•7H_2_O, 0.05 g FeSO_4_•7H_2_O, 3.0 g Na_2_CO_3_. Na_2_CO_3_ and NaCl (autoclaved separately). The temperature of growth was 35 °C.

*Parageobacillus thermantarcticus*^39,40^ a thermophilic species isolated from Mt. Melbourne, an active volcano in Antarctica. It is a Gram-positive, motile and spore forming rod. It was grown in YN medium contained the following components (per litre): 6 g yeast extract, 3 g NaCl at pH 5.6. The optimal growth temperature was 60 °C.

*Haloterrigena hispanica*^41^ an extremely halophilic archaea bacterium isolated in southern Spain from a salty lake in Fuente de Piedra. It was grown at 50 °C on DSM 372 medium contained the following components (per litre): 5.0 g yeast extract, 5.0 g casamino acids, 3.0 g trisodium citrate, 2.0 g KCl, 20 g MgSO_4_•7H_2_O, 200 g NaCl, 0.36 μg MnCl_2_•4H_2_O and 0.05 g FeSO_4_•7H_2_O at pH value 7.0.

*Geobacillus toebii* subspecies *decanicus*^42^ a Gram-positive, motile rod, isolated from hot composting process, was grown using TH medium contained the following components (per litre): 8 g peptone, 4 g yeast extract, 2 g NaCl, pH 7.0. The optimal growth temperature was 65 °C.

*Bacillus saliphilus*^43^, a haloalkaliphilic Gram-positive bacterium isolated from a mineral pool located in Campania region (southern Italy). It was grown at 37 °C in the medium A as described above but containing 160 g/l of NaCl instead of 100 g/l. *Nesterenkonia aurantiaca*^44^, a haloalkaliphilic and psycrophilic Gram-stain-positive actinobacterium isolated in Antarctica from soil collected in Cape King. It grew in medium A as described above but containing 20 g/l of NaCl instead of 100 g/l. The optimal growth temperature was 25 °C.

All the species investigated are summarized in Table 1 along with their isolation place and optimal parameters for growth.

In order to assess nitrate reduction activity, the corresponding optimal liquid growth media of the above microorganisms plus 0.1% (w/v) KNO_3_ (nitrate broth) were used.

10 ml of nitrate broth was inoculated with bacteria and incubated at the temperature required; after 24 h of incubation the cells were harvested and the supernatants with appropriate dilutions tested by the Aquamerck 11170 nitrate test (10–150 mg/l) according to the manufacturer’s instructions.

The results reported showed that *H. campaniensis* sp. nov., strain 5AG^T^ (DSM 15293^T^, ATCC BAA-966^T^) possesses comparatively a higher rapidity to reduce nitrate using the enzyme nitrate reductase. This microorganism was therefore selected to be applied as a potential bio-cleaning agent of nitrate efflorescence on stone surfaces.

### Cultural condition and viable count of the selected bio-cleaning bacterium *Halomonas campaniensis*

*H. campaniensis* was grown in medium A as reported above. Solid medium A contained 1.8% of bacteriological agar. *H. campaniensis* is able to grow at the temperature range of 10.0–43.0 °C and in the pH range 7.0–10.0 with an optimum at pH 9.0. It tolerates up to 16% NaCl with an optimum at 10% NaCl concentration.

Viable cell counts were performed by standard plate count method. Briefly, 100 µl of bacterial growth was used to prepare decimal dilutions, which were plated in duplicate with Plate Count agar. The average colony count of duplicate plates was used to calculate the Colony Forming Units (CFU)/ml.

### Preparation of stone samples artificially enriched with nitrate

NYT stone collected from a quarry located in Quarto, Naples, Italy was cut into samples of 5 × 5 × 1 cm (see Fig. 6a,b). The square tiles were artificially enriched with nitrate to simulate nitrate efflorescence on stone surface caused by salt capillary rise. Each stone sample was immersed at room temperature in 15 ml of KNO_3_ saturated solution deposited on Petri’s dish (8.0 cm diameter) and then kept in an oven at 60 °C for 48 h for drying. Nitrate was reasonably distributed over the surface of each stone sample. As no nitrate dispersion through salt deposition into Petri’s dish was observed, it can be assumed that each stone tile was enriched with 4.80 g of KNO_3_ crystallized both on the stone surface (efflorescence) and within (sub-efflorescence). The reproducibility of the method set up was therefore assumed acceptable.

The NYT samples enriched with nitrate were then used to assess the nitrate reducing efficiency of the extremophilic bacterium selected.

### Bio-cleaning treatments

Bacterial growth was obtained by inoculating *H. campaniensis* preculture (1:100) in 200 ml of medium A into a 0.5 l Erlenmeyer flask at 35 °C and 120 rpm for one night. After incubation, bacterial growth containing approximately 10^9^ CFU/ml was recovered by centrifugation at 10,000 rpm for 15 min.

Wet cell pellets were washed with sterile buffer solution RS (g/l): potassium chloride 0.150, sodium chloride 2.25, sodium bicarbonate 0.05, calcium chloride hexahydrate 0.12, pH 7.5. It was found that by adding 0.1 ml of sterile buffer solution to 150 mg of wet cell pellet a specimen was obtained that was easy enough to be handled and stiff enough to remain on the surface of stone. Such a finding induced to do not entrap bacterial cell by means of a delivery system as reported in literature^3,4,8^, at this step of our investigation at least. *H. campaniensis* specimens so achieved were applied to the surface of NYT samples using a sterile spatula as a uniform thin layer. 2% agar discs (prepared in distilled water supplemented with 2% (w/v) NaCl, autoclaved and placed in sterile Petri dish 5 cm diameter, 5 mm high) were used to cover the surface of treated (with microorganism) and untreated (without microorganism) NYT samples (see Fig. 6c,d). Subsequently all the samples were incubated under controlled conditions in a glass chamber (35 °C and 93% humidity). After incubation time, the agar disc and bacterial layer was removed from the stone surface with a sterile spatula and the bio-cleaning process monitored by FTIR analysis. Nitrate content on stone treated and untreated surfaces was assessed at the following incubation times (t): t 0 h, t 0.5 h, t 1.5 h, t 3.0 h, t 6.0 h, t 12 h.

In order to explore nitrate reducing ability of *H. campaniensis* in a real outdoor environment, treated and untreated NYT samples supplemented with agar discs were exposed outdoors in Pozzuoli, Naples, Italy (40°49′59″52N, 14°6′19″44E), 28 m above sea level for 3 h during October, 2017 protected through a film layer (see Fig. 6e and f).

It is well known that nitrate is highly soluble salt in water, thus to evaluate the real ability of *H. campaniensis* bacteria in nitrate bio-cleaning, nitrate determination was carried after incubation/treatment time without performing any water washing of the treated and untreated stone surfaces after removing the agar disc and bacterial layer with a spatula and agar disc respectively.

### Tests to assess nitrate bio-removal on stone surface after incubation/treatment

Preliminary investigation for assessing nitrate content on treated and untreated surfaces of NYT samples after incubation under controlled conditions was carried out with the Aquamerck nitrate test described above. 0.5 g of powder sample was obtained by uniformly scratching the outer surface layer of untreated and treated NYT with a blade. Appropriate dilutions were needed for the determination of nitrate.

A more careful determination of nitrate content was performed by means of FTIR spectroscopy operating in transmission mode. A Perkin Elmer Spectrum 100 spectrophotometer was used. The instrumental parameters adopted for the FTIR monitoring tests were as follows: resolution 4/cm, spectral range 8000–4000/cm. KBr classical pellet method was applied on powder samples of untreated and treated NYT stone. The weighing was carried out by means of an analytical balance Gibertini E 50 S (accuracy ± 0.0001 mg) and sample thickness measured through a caliber Quantumike – Mitutoyo (max 25.000 mm ± 0.001 mm). Statistical non-linear regression analysis was carried out on the results achieved by performing at least five treatments for each time point.

### Tests to assess nitrite formation on stone surface after incubation/treatment

The spectrophotometric determination of nitrite is based on the Griess diazotization reaction. Nitrite is detected and analyzed by formation of a red pink colour upon treatment of NO_2_^−^ containing sample with the Griess reagent (0.6% sulphanilic acid in HCl 2 N and 0.2% naphthylethylenediamine dihydrochloride, HCl 0.1 N) showing a characteristic absorption peak ranging between 520 nm and 540 nm. The standard solution of nitrite was prepared by dissolving 0.225 g of dried sodium nitrite (105 °C for 1 h) in 1000 ml of double distilled water. The nitrite content of the solution was 150 μg/ml. Reference curves was prepared in a range of concentrations from 3–30 μg NO_2_^−^ using 4 ml of Griess reagent in a final assay volume of 50 ml, which was protected from light. Absorbance at 520 nm was measured within 30 minutes at room temperature.

### Interaction between *Halomonas campaniensis* and real environments

Interaction between *H. campaniensis* and outdoor/indoor environments was investigated on samples of pristine NYT (2 × 2 cm^2^) untreated and treated with *H. campaniensis* exposed in Pozzuoli, Naples, Italy (40°49′59″52N, 14°6′19″44E) from 1 to 30 October 2017. The outdoor weather parameters are supplied by www.ilmeteo.it and the exposure was conducted as follows: all of the samples were exposed to the same conditions with uniform air access from all directions. Environmental parameters recorded are reported as follows: average temperature (°C) 18; minimum temperature (°C) 13.6; maximum temperature (°C) 22.6; relative humidity 65%; wind average rate 7.9 km/h; 2 rainy days; 2 stormy days and 2 foggy days. Indoor exposure was carried out in the ICB Microbiology lab located in Pozzuoli with an average temperature of 22 °C and a relative humidity ranging between 50% and 60%. All the stone samples, i.e without and with bacteria, were processed after 1–3–7–14–21–30 days as follows: the surface of all of the outdoor/indoor exposed NYT samples were washed with 10 ml of sterile medium A to recover bacteria and the wash solution was incubated for 24–48–72 h at 35 °C and 120 rpm in order to verify the growth of *H. campaniensis*.

The growth and morphology of microorganism were checked with by phase-contrast microscopy (Nikon 40×) and the Gram staining of cells was performed by using a Gram Staining Kit (Sigma-Aldrich), according to the manufacturer’s instructions. For a given time lapse three samples were observed at least.

### Tests to assess microorganism removal after washing

To remove residual cells (after taking out agar disc and bacterial layer with sterile spatula) stone treated surfaces were carefully washed with a sterile sponge impregnated with sterile distilled water at 40 °C. Removal efficiency of all the cells including non-viable cells was then investigated by means of Thermogravimetric Analysis (TGA), being the thermogravimetry a technique useful to identify organic materials by measuring the temperature of bond scissions in inert atmospheres or of oxidation in air or oxygen. TGA analysis was carried out on 20 mg of sample achieved by uniformly scratching the outer surface layer of NYT stone with a blade and weighed out using a Mettler thermobalance TGS-2; the resolution of the mass decrement changes was 0.0001 mg. NYT samples treated for 3 h, without and after washing, underwent the following thermal run: samples were heated under nitrogen atmosphere at a rate of 30 °C/min from 30 °C to 200 °C, kept at 200 °C for 30 min, cooled to 30 °C at a cooling rate of 30 °C/min and then heated from 30 °C up to 800 °C at a heating rate of 5 °C/min. Pristine tuff, Potassium nitrate and *H. campaniensis* specimens were also analyzed in the same conditions.

Moreover, the presence of residual viable cells on the surfaces of the NYT samples treated with *H. campaniensis*, after washing with water was confirmed by standard a plate count method: 100 µl of the last washing water were spread on agar plate and incubated for 3 days at 35 °C; blank controls consisting of stone samples without bacteria.

## Supplementary information

Supplementary information
