# Supplementary material for: Bio-cleaning of nitrate salt efflorescence on stone samples using extremophilic bacteria
Source: Sci Rep. 2019 Feb 7;9:1668. doi: 10.1038/s41598-018-38187-x (PMC6367513; doi:10.1038/s41598-018-38187-x)
Supplement: Supplementary file 1 — Supplementary information [file 41598_2018_38187_MOESM1_ESM.pdf]

Supplementary Information

Bio-cleaning of nitrate salt efflorescence on stone samples using extremophilic bacteria

Ida Romano[a,] Mario Abbate[b], Annarita Poli[a] and Loredana D'Orazio[b]*,

[a] Institute of Biomolecular Chemistry of Consiglio Nazionale delle Ricerche, 80078 Pozzuoli, Naples, Italy

[b] Institute for Polymers, Composites and Biomaterials of Consiglio Nazionale delle Ricerche, 80078 Pozzuoli, Naples, Italy

*Corresponding author

e-mail address: loredana.dorazio@ipcb.cnr.it

Fig. S1:

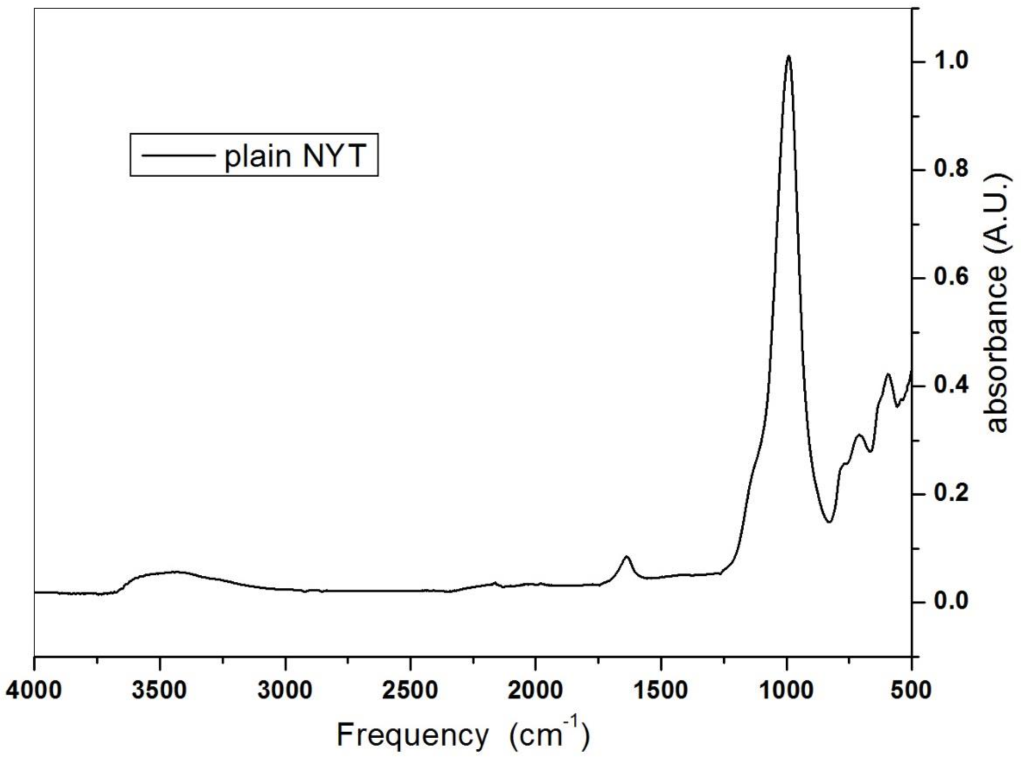

Figure S1: FTIR spectrum exhibited by plain NYT.

Fig. S2:

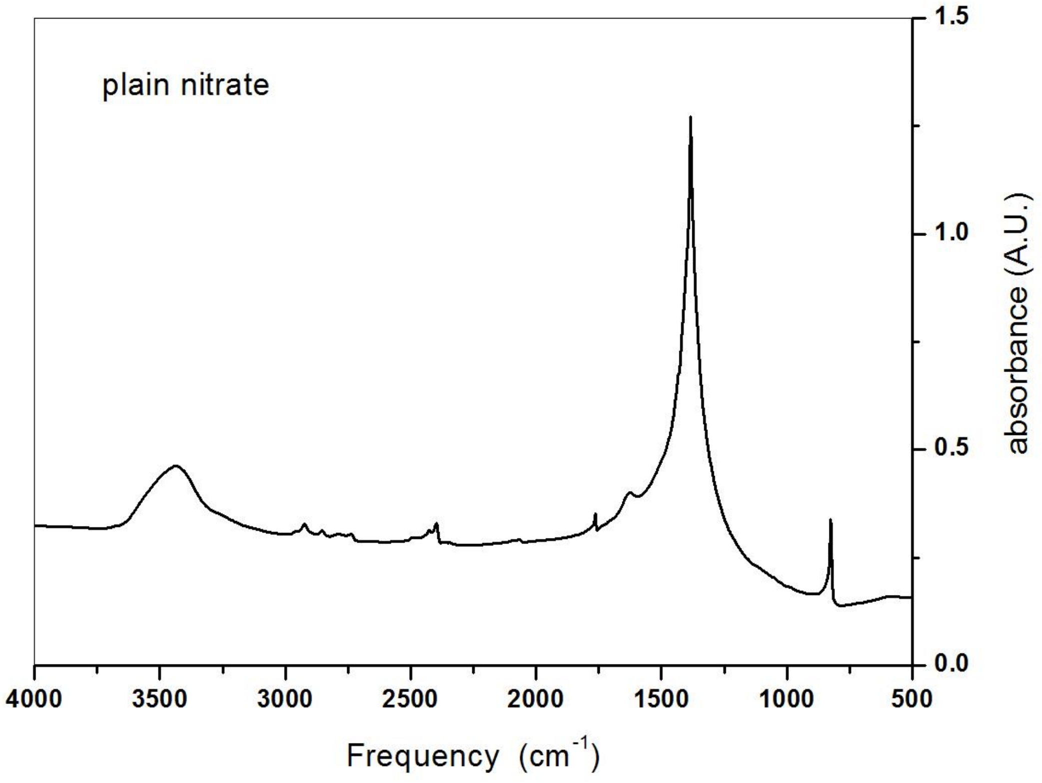

Figure S2: FTIR spectrum exhibited by plain Potassium nitrate.

Fig. S3:

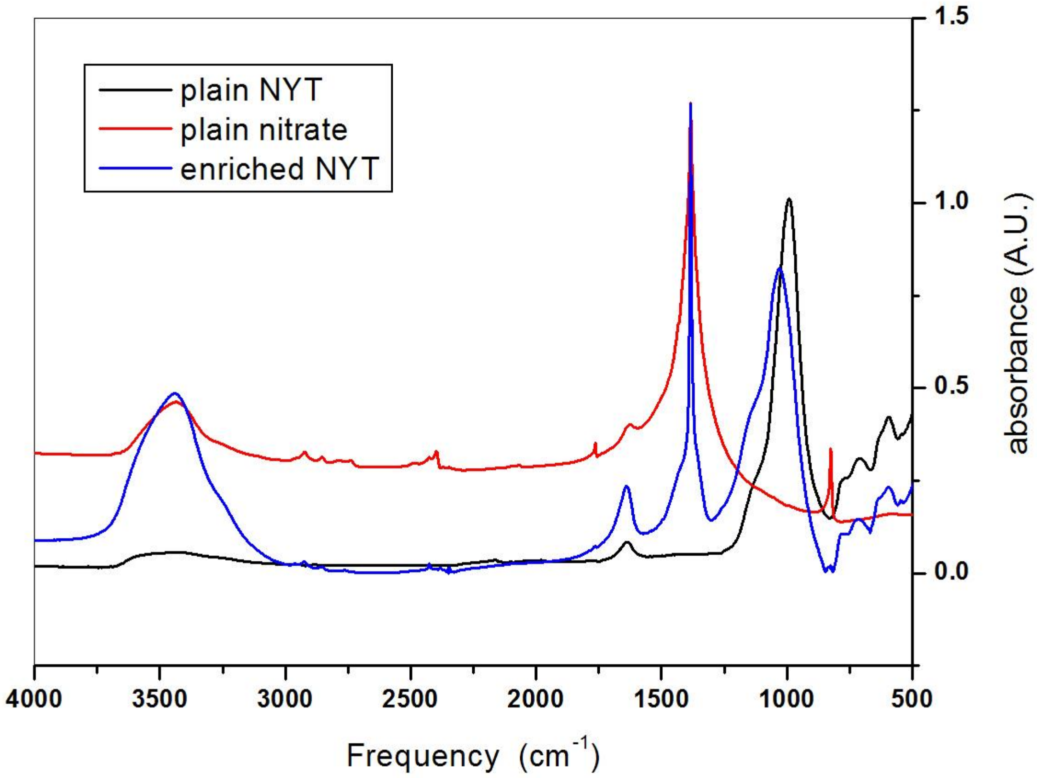

Figure S3: FTIR spectrum shown by nitrate enriched NYT samples.

Fig. S4:

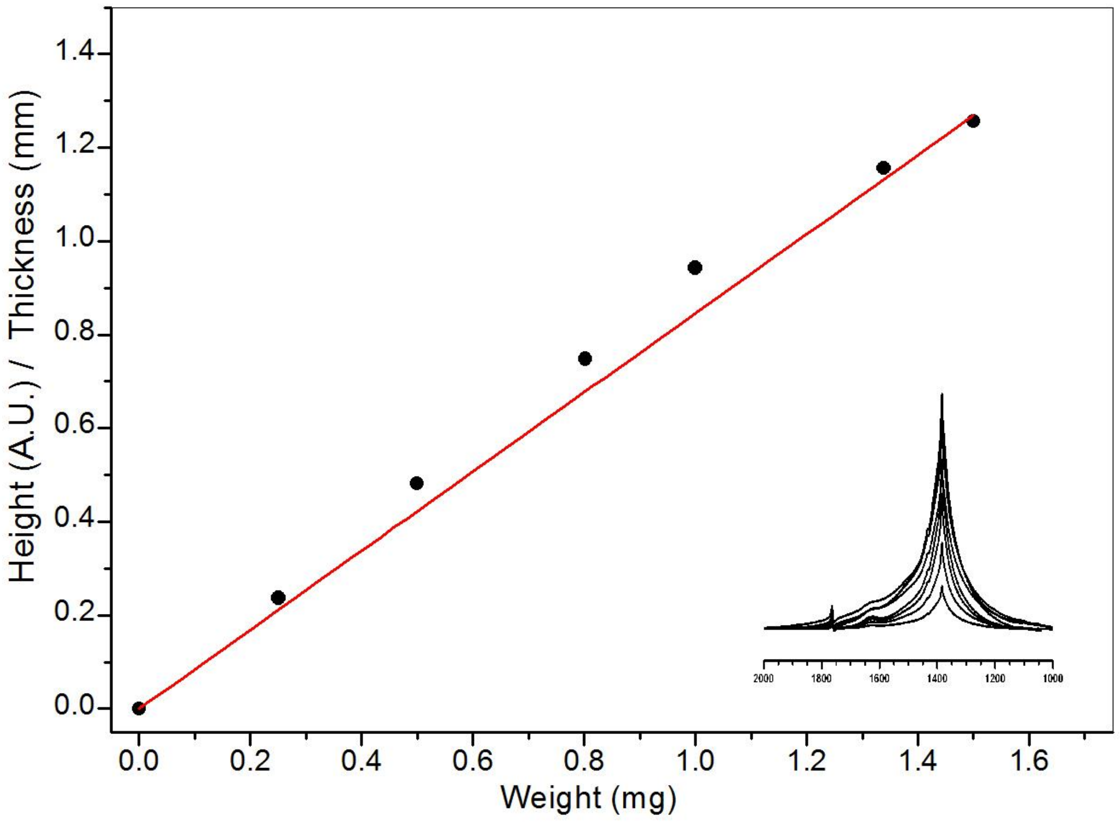

Figure S4: Calibration plot constructed plotting the absorbance values recorded at 1385 cm^{-1}normalized for the sample thickness against nitrate amount.

Fig. S5:

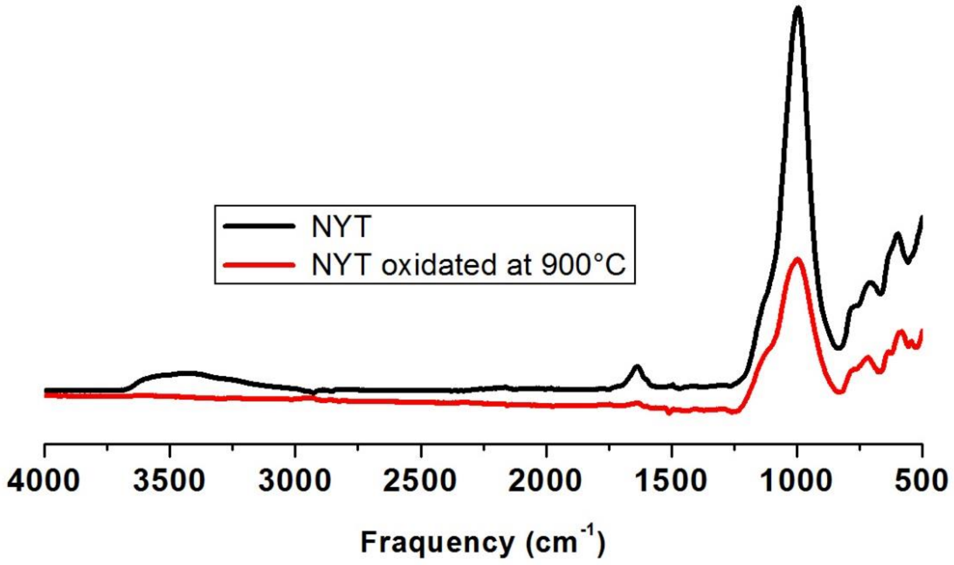

Figure S5: FTIR spectrum of NYT samples heated to 900 °C.

Table S1:

Assignment	Frequency
ν_2	824 cm^{-1} (m, sp)
ν_3	1385 cm^{-1} (vs)
ν_4	1767 cm^{-1} (vw)

m = medium, sp = sharp, vs = very strong; vw = very weak: peak relative intensity.
[a]Main assignments: ν_2 = out of plane bending, ν_3 = doubly degenerate anti-symmetric stretching, ν_4 = doubly degenerate planar bending.

Table S1
Frequencies and assignments of the main absorption bands of KNO_3 phase[a].
